# A Decision Tree Analysis of Diabetic Foot Amputation Risk in Indian Patients

**DOI:** 10.3389/fendo.2017.00025

**Published:** 2017-02-17

**Authors:** Prasad Umesh Kasbekar, Pranay Goel, Shailaja Prakash Jadhav

**Affiliations:** ^1^Department of General Surgery, B.J. Government Medical College, Pune, India; ^2^Indian Institute of Science Education and Research, Pune, India

**Keywords:** diabetic foot, amputation risk score, C5 classifier, prognostic indicators, ulcer prognosis

## Abstract

**Aim:**

The aim of this study is to create an evidence-based tool that guides the risk of amputation in diabetic foot patients.

**Materials and methods:**

Hospital records of 301 diabetic foot patients were examined retrospectively for explanatory variables of foot amputation decisions. The study included all patients with a lower limb ulcer with a known history of diabetes mellitus or those diagnosed post-admission. The dataset was analyzed, and a risk scoring system was constructed using the decision tree algorithm, C5.0. Two classifiers, one simple and another complex, were constructed for predicting amputation outcome.

**Results and discussion:**

Based on our evaluation, the most influential predictors for a decision to amputate are Doppler flow measurements and the Wagner grading of the ulceration. The simple classifier uses just these two parameters in determining risk. The results obtained show an accuracy of 96.4% in the primary group and an accuracy of 94% in the test group. The second classifier is a more complex computer-derived construct that showed 100% accuracy in the principle group and an accuracy of 96% during testing.

**Conclusion:**

In the present era of *precision medicine*, these two classifiers act as an accurate guide to the prognosis of the limb in patients with diabetic foot and can predict the risk of future amputation.

Diabetic foot disease is a well-known complication of diabetes; its incidence rate is greater than all other complications combined (retinopathy, nephropathy, and others) (1, 2). One of the major predicaments faced by a doctor during treatment is determining the point of no return, i.e., the time for amputation. Our study focuses on attempting to determine those factors which affect the outcome of amputation in diabetic foot patients and create a prognostic scoring program to assess the degree of inevitability involved in amputation in the diabetic foot patient.

Various classification systems have previously been proposed to estimate the risk of amputation in patients with diabetic foot disease. However, no system has been formally adopted as routine methodology for risk assessment. Early classification systems included the Meggitt–Wagner (3) system and the SINBAD classification (4). The International Working Group of the Diabetic Foot (IWGDF) in 2004 developed the PEDIS classification (5), which has been widely regarded as a baseline, consensus model. The PEDIS system ranks ulcer-related complications in terms of perfusion (blood supply), extent (size), depth, infection, and sensations as the parameters to assess diabetic foot risk. Recent studies by Quilici et al. (6) and Won et al. (7) have stressed not only the severity of ulcer but also the state of peripheral vascular disease in the prognosis of diabetic foot. Interestingly, neither blood sugar status nor diabetes controls feature directly in their analysis, however, long-term antibiotic usage did adversely influence outcome. Pickwell et al. (8) recently developed a risk stratification algorithm with greater sensitivity than the current PEDIS system: in a multicenter study of the IWGDF, they prospectively analyzed 575 patients and found periwound edema, foul smell, purulent exudates, deep ulcer, a positive probe-to-bone test, pretibial edema, fever, and increased C-reactive protein as risk factors for amputation; a risk scoring system was developed for predicting risk of amputation using these variables. Other attempts to determine the risk of amputation are due to Lipsky et al. (9) and Beckert et al. (10). In the study of Pemayun et al. (11), HbA1C levels greater than 8, the presence of peripheral arterial disease, hypertriglyceridemia, and hypertension are independent risk factors for amputation.

A comparative study by Jeon et al. (12) of five different classification systems showed the Meggitt–Wagner system to be the most accurate in predicting the risk for lower extremity amputation. Monteiro-Soares et al. (13) conducted a meta-analysis of all the prevalent diabetic foot amputation risk classification systems available (15 in all) prior to 2013 and found Meggitt–Wagner, S(AD)SAD, and the Texas University Classification to be the most validated and accurate of the available systems. Monteiro-Soares and Dinis-Ribeiro (14) went on to develop their own DIAFORA tool (using a cohort of 293 patients); their algorithm comprises of eight variables used to construct point scoring system for amputation.

In diabetes foot healthcare in India, there is a similar need to develop a system that can be validated and standardized for this population. There are significant challenges in the management and care of these patients, including a large patient load, low socioeconomic standards, and patient illiteracy which, in turn, lead to poor patient compliance and follow-up. Developing an algorithm for assessing amputation risk can be useful not only in prognosticating the limb and triaging patients of diabetic foot disease but also in the individualization of treatment meted to patients. It is useful to note that one widely used parameter in practice at both secondary and tertiary health-care centers is *Doppler flow* analysis. We therefore expect that incorporating Doppler readings into amputation risk assessment is likely to lead to a greater adoption, reproducibility, and better standardization in this population.

The methodology we use here is that of using a *decision tree* (15) to predict the risk of amputation. Decision trees are a very popular technique for analyzing medical outcomes because they are structured to facilitate the user in comparing the prediction of outcomes under different simulated test scenarios. A well-known example of this methodology is the type 2 diabetes risk self-assessment test of the American Diabetes Association: http://www.diabetes.org/are-you-at-risk/diabetes-risk-test/. A user answers a few questions posed by a computer system, which analyses this combination of risk factors and returns the likelihood that they have diabetes.

To develop a decision tree, various patient and disease characteristics are fed into a supervised machine learning algorithm (a popular algorithm is called C5.0), which examines the data and produces a flowchart; this can then be used to classify a new patient as an (un)likely candidate for amputation. It is useful to note that these algorithms typically use only a subset of the full spectrum of clinical test variables in the flowchart. In other words, the goal is to discover those particular *combinations of variables* that are the most effective in terms of the ability to correctly predict the likelihood of the outcome of interest. The direct usability of risk decision trees makes them ideally suited for use in clinical environments. To the best of our knowledge, the present study is a novel approach in diabetic foot amputation risk classification.

## Research Designs and Methods

### Patient Data

Hospital records of 301 diabetic foot patients, some of whom underwent amputation surgery, during the period June 2011 through June 2013, were examined retrospectively. The patients comprised locals, primarily of Indian origin, who came to our hospital seeking treatment. A total of 203 patients were known diabetics, while 98 were diagnosed as diabetics on admission. All patients were evaluated by recording a detailed history, clinical examination, and other necessary investigations. The end point of the evaluation was complete healing of wound or skin grafting in the conserved patients, or amputation with the healing of stump in the amputated patients. In total, 83 patients underwent amputation, while 218 were managed successfully by conservative treatment.

The inclusion criteria for the present study were as follows: all patients with a lower limb ulcer, a known history of diabetes mellitus, or those diagnosed post-admission. All ages were included. The exclusion criteria were as follows: patients who died, or withdrew during the course of their treatment, or were lost to follow-up prior to reaching the end-points of evaluation.

Management decisions regarding the patients were approved by the respective hospital Unit heads-of-the-department under which the patients were admitted. Unit heads and respective unit members were blind to the study. Each Unit was headed by a Professor or Associate Professor with a minimum of 9 years of working experience. The investigator’s role was purely observational; no active intervention was carried out on the patient or the decision making during the study.

### Clinical, Explanatory Variables

Patient history included the following: the presence of diabetes, whether treatment included insulin or other oral hypoglycemic agents, the duration of leg symptoms (pain, trauma, ulcer, blackening or reduced movements, whichever was first noticed by the patient, whether patient took or was taking prior treatment for same), history of regular smoking or alcohol intake, any other comorbidities, age, sex, and weight.

Clinical examination focused mainly on the limb ulcer and its grading as per the Wagner–Meggitt classification (3) along with the examination for other co-existing comorbidities, if any.

Investigations carried out were as follows: blood hemoglobin levels, serum creatinine, blood prothrombin time, serum albumin, serum bilirubin, random blood glucose level on admission, and glycosylated hemoglobin level (HbA1C) levels. Other investigations included the following: a skiagram (X-ray) of the affected foot, pus culture and sensitivity examination of the wound, and an arterial Doppler (duplex scan) finding of the limb. Flow in the limb was recorded as absent, monophasic, biphasic, triphasic, or normal based on their waveforms on the duplex scan (16, 17). Absent flow indicated the complete absence of flow in all vessels distal to the point of involvement; however, the collateral formation was not taken into account in the Doppler findings.

These parameters were registered at the time of the first contact with the patients. Subsequent changes post-treatment initiation were not considered as our study wanted to focus on the prognosis of patients at first contact, prior to initiation of any treatment at our institute, regardless of the treatment taken outside. The duration of patient stay in our institute was considered to see if it had any impact on the final outcome of the limb. The patients were followed up for a period of 1 year to determine the success of the treatment given as well as their adherence to the diabetes care.

### Analytical Methods

The patient dataset was randomly divided into two groups: data from 250 patients were retained for training the decision tree algorithms. The predictions of the classifier were then tested on the remaining 51 patients. In this way, bias is eliminated in the validation of the learnt classifier. The dataset used and the corresponding scripts are provided in Supplementary Material.

The decision tree was constructed using R and the CARET package (18) implementation of the algorithm, C5.0. We carried out two analyses of the data and constructed two classifiers.

### A Simple Classification and Regression Tree

The first is a single *C5.0 tree* that predicts amputation on the basis of just two variables: arterial Doppler flow and ulcer grade.

### A Boosted Classification and Regression Tree

The other classifier is a *boosted C5.0 tree ensemble*. The ensemble algorithm weighs in the recommendations of a “committee” of 10 separate decision trees to make a final prediction. It thus uses a more comprehensive panel of variables to make a decision.

## Results

### A Single C5.0 Tree

The first prediction algorithm is a C5.0 decision that represents a “coarse” view of the predictions. The following are the performance metrics of this algorithm.

### Training the Classifier

**Figure 1:**
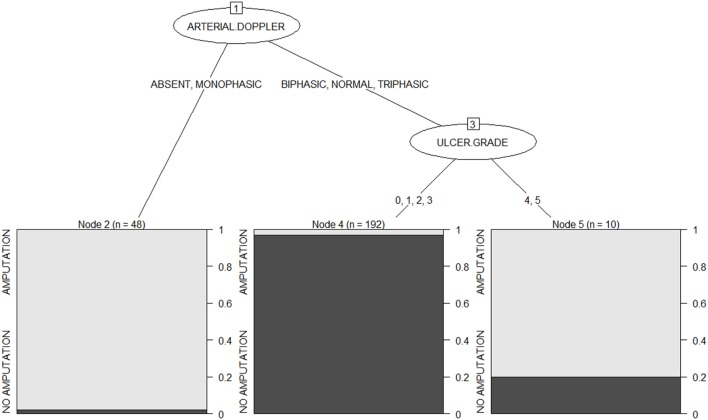
**A simple C5.0 decision tree for predicting amputation risk**.

The option of “winnowing” available with C5.0 results in a small tree of depth 3 is shown in Figure 1. This can be interpreted as follows: the first predictor of amputation is the ulcer grade. Those with absent or monophasic peripheral flow on Doppler will have a very high risk of amputation in the future. While those with normal, triphasic, or biphasic flow should have their ulcer grade examined. Grades 4 and 5 limbs have an 80% chance of amputation, while those with grade 0, 1, 2, or 3 limbs have more than 95% chance of conservation.

The confusion matrix obtained during the training phase is shown in Table 1. The *error rate* is low, 3.6% (i.e., 9 cases out of 250 are misclassified).

**Table 1:** **The confusion matrix obtained as a result of training the simple C5.0 decision tree**.

		Predicted management	
		Amputation	No amputation
**Actual management**	**Amputation**	55	6
	**No amputation**	3	186

The “attribute usage” in the decision tree is as follows: the values of arterial Doppler in 100.00% of cases and the values of ulcer grade in 80.80% of the data were used in classification. That is, arterial Doppler played a role in 100% of cases, while ulcer grade influenced the outcome in 80.8% of cases.

### Testing the Performance of the Classifier

When tested with an independent dataset of 51 cases, that is, those who were not used for training the decision tree, the accuracy of the algorithm is 94%, with a kappa of 0.88. (The kappa statistic is a fraction between 0 and 1 that compares the accuracy of the classifier to that of a random system; a higher kappa is indicative of good performance of the classifier.) The confusion matrix as a result of testing is shown in Table 2.

**Table 2:** **The confusion matrix obtained as a result of testing the simple C5.0 decision tree**.

		Predicted management	
		Amputation	No amputation
**Actual management**	**Amputation**	20	2
	**No amputation**	1	28

The simple classifier system leaves several questions unanswered as to the role of the other variables in the prediction of outcome in diabetic foot disease. We therefore attempted to factor in the other variables to ask if they have a role in the outcome of diabetic foot disease. Thus, an ensemble predictor was created, which includes a greater number of explanatory variables in determining the outcome. Not only is it satisfying that a number of clinically relevant variables are used in the analysis but this also results in greater prediction accuracy.

### The Boosted C5.0 Tree Ensemble

#### Training the Classifier

The training phase results in a perfect classification of all cases. That confusion matrix is shown in Table 3. Ten trees of different sizes and accuracies (not shown individually) are used in the ensemble; the net error is 0, as shown in Table 4. Attribute usage shows a comprehensive panel of the variables that are included in the classifier, as shown in Table 5.

**Table 3:** **The confusion matrix obtained as a result of training the ensemble C5.0 boosted decision trees**.

		Predicted management	
		Amputation	No amputation
**Actual management**	**Amputation**	61	0
	**No amputation**	0	189

**Table 4:** **The accuracy obtained as a result of training the ensemble of C5.0 boosted decision trees**.

Trial no.	Decision tree size	Errors
0	11	6 (2.4%)
1	6	16 (6.4%)
2	4	49 (19.6%)
3	7	17 (6.8%)
4	8	11 (4.4%)
5	9	8 (3.2%)
6	9	16 (6.4%)
7	7	21 (8.4%)
8	8	28 (11.2%)
9	14	4 (1.6%)
Boosted ensemble		0 (0.0%)

*Notice the error rate decreases as more decision trees are inducted into the ensemble, with a final (training) error of 0, i.e., resulting in a perfect classification of all training examples*.

**Table 5:** **The attribute usage in the ensemble C5.0 boosted decision trees**.

100.00%	Days admitted
100.00%	Ulcer grade: 1
100.00%	Ulcer grade: 5
100.00%	Foot X-ray: Normal
100.00%	Doppler: Monophasic
100.00%	Doppler: Triphasic
99.60%	HbA1c
99.20%	Ulcer grade: 4
98.80%	Doppler: Normal
96.40%	Creatinine
89.60%	Duration of symptoms
84.00%	Other comorbidities? Yes
63.20%	Ulcer grade: 2
57.20%	Age
43.60%	Prothrombin time
36.00%	BSL on admission
34.40%	History of diabetes: Yes
32.00%	Albumin
32.00%	Doppler: Biphasic
20.80%	Culture report: No growth
18.80%	Hemoglobin
14.80%	Bilirubin
10.80%	Ulcer grade: 3
8.00%	Weight

#### Testing the Classifier

The confusion matrix in the testing phase is shown in Table 6. The accuracy is 96% with a 95% CI: 0.8654, 0.9952. Kappa is 0.92.

**Table 6:** **The confusion matrix obtained as a result of testing the ensemble C5.0 boosted decision trees**.

		Predicted management	
		Amputation	No amputation
**Actual management**	**Amputation**	20	2
	**No amputation**	0	29

## Discussion

We have created two machine-learnt classification algorithms for evaluating the risk of amputation of a diabetic foot. One of these is simpler to use: its predictive capacity involves only two explanatory variables, namely, Doppler flow reading and ulcer gradation. The second classifier is more complex and requires using a computer at hand to evaluate predictions. Both classifiers are highly efficient, achieving accuracy rates upwards of 90% when tested on a dataset different from the one used to train them. The latter classifier uses a more comprehensive panel of clinical variables in the analysis, and its prediction capacity is slightly better than that of the former, which compensates for the lack of “human readability” of the algorithm.

In terms of comparing the predictive ability of the two classifiers, the ensemble method is likely to perform slightly better of the two. Nonetheless, some insights can be obtained using the simple tree. Some cases are relatively easier to diagnose; such cases are evident from the simple tree: these are the cases when Doppler flow is either absent or monophasic, or when ulcer grade is 0, 1, 2, or 3. For the more ambiguous cases—or for greater confidence in the prediction, in general—it is perhaps better to rely on the prediction from the ensemble method.

From the above two algorithms, we conclude that the Doppler status of flow in the affected limb and the ulcer grade clinically are the most important factors in determining the prognosis of the limb. Those patients with an absent or monophasic flow in the limb are at highest risk of amputation. As blood supply to the limb reduces, there is less perfusion and lesser chance of wound healing which ultimately would result in complications and eventually amputation. With better peripheral flow in the limb, that is, biphasic, triphasic, or normal Doppler flow, the chances of amputation reduce rapidly. Even those with Wagner classification grade 4 or 5 limbs show a 20% chance of limb conservation. High-grade clinical classification with good flow may indicate a microvascular abnormality and aggressive management of these limbs may end up in limb salvage.

## Conclusion

In keeping with the tenets of precision medicine, the objective of our study was to create a classification system to ascertain the prognosis of the limb in diabetic foot patients. Its intended utility is to help doctors and patients in achieving a clearer understanding of the state of the disease in the patient and guide further management. This is especially required in countries such as India where excessive patient load, poor follow-up, and poor compliance to treatment are major drawbacks in healthcare.

We have created two predictive classifiers of amputation risk assessment. The simple classifier is very good as a broad-based classifier to understand the approximate prognosis of the patient’s limb. Many cases, however, may not be simple to prognosticate: for example, those patients with ulcer grade 3, 4, or 5 and good Doppler flow or those with ulcer grade 0, 1, or 2 and monophasic or no flow on Doppler; in these cases, a more comprehensive classifier is needed in order to estimate the outcome of the limb, using multiple characteristics. Our second classifier achieves an even greater accuracy in our base as well as test subjects. This algorithm has shown excellent accuracy: 100% accuracy in the training group and greater than 96% accuracy in the test group, with only two cases being misclassified. As the algorithm is complex, however, predictions can only be done *via* a computer-based system. This is thus advised for the doubtful cases, such as those cases of normal, triphasic or biphasic flow in the major vessels of the limb with an ulcer grade 3, 4, or 5. Such cases can otherwise prove a clinical dilemma: the foot exhibits good limb blood flow; however, it simultaneously presents with a clinically advanced ulcer.

Our method can be used not only in a personalized manner for management decisions but also for triage of patients based on risk. Further, it can be useful in a scalable manner. We caution, however, that we envisage our classifier be used as evidence-based tool to aid decisions, and not as an absolute, independent tool for amputation. It can also be used as a basis for further studies in the field.

## Ethics Statement

The present study is a mathematical and statistical analysis of a diabetic foot patient database that was collated as part of a thesis submitted by Prasad Umesh Kasbekar to B.J. Medical College, Pune, titled “Evaluation of Diabetic Foot Ulcer Patients and Development of a Probable Risk Score for Amputation of the Extremity.” Detailed consent was obtained from patients about the nature of the study and the use of their data. The Ethics Committee of B.J. Medical College, Pune, approved that study. This manuscript is a new analysis of those data. In particular, we work with de-identified data from the repository. No new data have been collected for this manuscript, and no interventions have been performed; only analytical results are presented in the manuscript. A separate ethics review process was not sought for this study.

## Author Contributions

PK—case finding and follow-up, data collection, analysis of data, and manuscript wording. PG—data analysis, creation of decision tree, and manuscript wording. SJ—case finding and follow-up, and manuscript wording.

## Conflict of Interest Statement

The authors declare that the research was conducted in the absence of any commercial or financial relationships that could be construed as a potential conflict of interest.
